# Neural growth hormone: regional regulation by estradiol and/or sex chromosome complement in male and female mice

**DOI:** 10.1186/s13293-015-0026-x

**Published:** 2015-04-28

**Authors:** Kayla M Quinnies, Paul J Bonthuis, Erin P Harris, Savera RJ Shetty, Emilie F Rissman

**Affiliations:** Department of Biochemistry and Molecular Genetics, University of Virginia School of Medicine, Charlottesville, VA 22908 USA; Neuroscience Graduate Program, University of Virginia School of Medicine, Charlottesville, VA 22908 USA; Department of Neurobiology and Anatomy, University of Utah, 20 North 1900 East, Salt Lake City, UT 84132-3401 USA; Department of Biological Sciences, North Carolina State University, Raleigh, NC 27695 USA

**Keywords:** Growth hormone, Sex differences, Estradiol, Sex chromosomes, Hypothalamus, Cerebellum, Growth hormone-releasing hormone, Obesity

## Abstract

**Background:**

Sex differences in pituitary growth hormone (GH) are well documented and coordinate maturation and growth. GH and its receptor are also produced in the brain where they may impact cognitive function and synaptic plasticity, and estradiol produces *Gh* sex differences in rat hippocampus. In mice, circulating estradiol increases *Gh* mRNA in female but not in male medial preoptic area (mPOA); therefore, additional factors regulate sexually dimorphic *Gh* expression in the brain. Thus, we hypothesized that sex chromosomes interact with estradiol to promote sex differences in GH. Here, we assessed the contributions of both estradiol and sex chromosome complement on *Gh* mRNA levels in three large brain regions: the hippocampus, hypothalamus, and cerebellum.

**Methods:**

We used the four core genotypes (FCG) mice, which uncouple effects of sex chromosomes and gonadal sex. The FCG model has a deletion of the sex-determining region on the Y chromosome (*Sry*) and transgenic insertion of *Sry* on an autosome. Adult FCG mice were gonadectomized and given either a blank Silastic implant or an implant containing 17β-estradiol. Significant differences in GH protein and mRNA were attributed to estradiol replacement, gonadal sex, sex chromosome complement, and their interactions, which were assessed by ANOVA and planned comparisons.

**Results:**

Estradiol increased *Gh* mRNA in the cerebellum and hippocampus, regardless of sex chromosome complement or gonadal sex. In contrast, in the hypothalamus, females had higher *Gh* mRNA than males, and XY females had more *Gh* mRNA than XY males and XX females. This same pattern was observed for GH protein. Because the differences in *Gh* mRNA in the hypothalamus did not replicate prior studies using other mouse models and tissue from mPOA or arcuate nucleus, we examined GH protein in the arcuate, a subdivision of the hypothalamus. Like the previous reports, and in contrast to the entire hypothalamus, a sex chromosome complement effect showed that XX mice had more GH than XY in the arcuate.

**Conclusions:**

Sex chromosome complement regulates GH in some but not all brain areas, and within the hypothalamus, sex chromosomes have cell-specific actions on GH. Thus, sex chromosome complement and estradiol both contribute to GH sex differences in the brain.

## Background

Growth hormone (GH) is synthesized in the pituitary and the brain [1,2]. Notably, like other anterior pituitary hormones, the secretion of GH is pulsatile and controlled by metabolic and neuroendocrine mechanisms [3-5]. There are long-established and well-documented sex differences in the frequency and amplitude of these GH pulses beginning during the peripubertal period in many mammalian species, including humans [6]. GH is also produced in the brain, where its distribution is extensive. GH receptors are present throughout the brain, and animal and human studies have implicated central GH signaling in neural functions [7]. For example, GH levels influence or are correlated with cognitive performance [8] and enhance excitatory hippocampal synaptic transmission [9]. GH also has neuroprotective effects, protecting from age-related hippocampal deficits in plasticity and learning [10] and against cerebellar damage following ischemic injury [11]. Additionally, GH has been broadly implicated in aging and neurogenesis, as neural precursor cells in the subventricular zone proliferate in response to central GH infusion [12]. Feeding behavior has also been linked to GH [13], and GH expression, in the hypothalamus specifically, is associated with sexually dimorphic body weight differences [14].

Regulation of GH differs by brain region. For example, in the hippocampus, *Gh* mRNA levels are lower prior to puberty and increased in post pubertal rats [15]. Also, *Gh* mRNA and protein expression are noted in many brain regions and known to be sexually dimorphic (females have higher mRNA expression and protein levels than males) in the hippocampus, preoptic area of the hypothalamus (mPOA), and arcuate nucleus in mice [16]. While sex differences in GH levels and release have been documented, the mechanisms that regulate these sex differences are not well explored.

Estradiol modulates GH protein and *Gh* mRNA expression in the rat hippocampus. In females, GH protein levels in the hippocampus are highest during estrus, when estradiol levels are elevated. In addition, ovariectomized rats express low levels of *Gh* mRNA and protein which increase following estradiol treatment [15]. In mice, the arcuate nucleus and the mPOA express sexually dimorphic *Gh* mRNA with females having higher levels than males. After gonadectomy, *Gh* mRNA decreases in females but not in males. Interestingly, castration actually increases *Gh* expression in the mPOA. Neurons containing GH protein also contain estrogen receptor alpha, and the anti-estrogen tamoxifen blocks the effects of estradiol on GH in females [16]. Thus, estradiol may regulate GH in females but not in males. In addition, the numbers of X chromosomes have a direct effect on *Gh* mRNA in the mouse mPOA (animals with two X chromosomes have higher levels than those with one X), and two genes on the X chromosome known to escape X inactivation are correlated with *Gh* expression in this region [14].

Taken together, existing data suggest that both estradiol and sex chromosome complement have actions on *Gh* mRNA and protein in a region-specific manner in the brain. Given the broad implications and regional specificity of GH, its regulation is important to assess. In this report, we tested these two factors simultaneously in three diverse brain regions: the hypothalamus, hippocampus, and cerebellum. We used the four core genotypes (FCG) mice, which have been previously utilized to compare sex chromosome versus gonadal sex as sources of sex differences [17,18]. In the FCG mice, gonadal sex is unlinked from the sex chromosomes by deletion of the sex-determining region on the Y chromosome (*Sry*) and transgenic insertion of *Sry* on an autosome [19]. The FCG cross produces four types of offspring: normal females with ovaries and XX chromosome genotype (XXF), females with ovaries and XY genotype (XYF), males with testes and XY genotype (XYM), and males with testes and XX genotype (XX males). To manipulate estradiol, adult gonadectomized mice were treated with chronic implants containing estradiol or no hormone. We report that estradiol treatment increased *Gh* mRNA significantly in the hippocampus and cerebellum. Sex chromosome complement was a factor only in the hypothalamus where XY females had the highest levels of GH protein and mRNA. Because the sex chromosome complement effect was in the opposite direction from our past reports in wild type and another sex chromosome mutant mouse model, we quantified GH protein in the arcuate nucleus. We replicated our previous finding that XX mice have more GH than XY mice in the arcuate, regardless of gonadal sex.

## Methods

### Animals

All procedures were approved by and conducted in accordance with the University of Virginia Animal Care and Use Committee guidelines. The mice used for all experiments were the FCG mice on a C57BL/6J background. The FCG mice are XX females, XY females, XX males, and XY males [18]. Mice were maintained on a 12:12 light cycle (lights on at 1:00 pm). Animals had access to water and food (# 7912 from Harlan Teklad, Madison, WI) *ad libitum*.

### Gonadectomy and hormone replacement

All mice between 75 and 85 days of age were gonadectomized. At the time of surgery, each mouse received a subcutaneous implant made of a 5-mm Silastic tube (Dow Corning, Corp., Midland, MI; 1.98 mm inner diameter × 3.18 mm outer diameter). Implants were either filled with 2 mg/ml 17β-estradiol benzoate in sesame oil (25 μl) or empty. During the surgery, mice were anesthetized with isoflurane. Following surgery, mice were given 0.9% sodium chloride subcutaneously and 0.25% bupivacaine as an analgesic and individually housed. All eight groups contained at least seven animals at the time of surgery.

### Tissue collection

Animals were anesthetized with isoflurane and killed by cervical dislocation 3 weeks after surgery. Brains were then collected and quickly free hand dissected on ice. The cerebellum was gently separated from the inferior colliculi and brainstem, and the hypothalamus was removed from the ventral surface of the brain by gently separating it from the cerebral hemispheres, brainstem, and the optic chiasm. The hippocampus was carefully separated from the cortex, hindbrain, and diencephalon by removing the cerebral cortex with an incision at the end of the hemisphere and one 2 mm rostral to the first incision. Next, the cortex was removed, which revealed the hippocampus. All tissue was rapidly frozen following dissection to preserve for RNA extraction. Estradiol-implanted animals that were designated for Western blots had the whole brain removed and rapidly frozen on dry ice.

### Quantitative real-time PCR

RNA was isolated from the brain tissue (Qiagen RNeasy Kit), and cDNA was generated from RNA by reverse transcription with Applied Biosciences High Capacity cDNA Reverse Transcription Kit. Real-time PCR was performed using the Applied Biosystems StepOne Plus for SYBR Green-based detection with Fast SYBR® Green Master Mix. Biological replicate samples of 5 ng were run in triplicate, and the average was used for data analysis. Quantification of *Gh* (F: 5′-AGGCCCAGCAGAGAACCGACA, R: 5′-ACGGTCCGAGGTGCCGAACA; source sequences: AK019954, AK030419, AL604045, BB024006, Consensus CDS: CCDS25554.1, UniProtKB/Swiss-Prot: P06880) gene expression levels was calculated based on the threshold cycle for each well using the provided software and normalized to *B2M* (F: 5′-GGCTCACACTGAATTCACCCCCAC, R: 5′-ACATGTCTCGATCCCAGTAGACGGT; source sequence: AK019389, Consensus CDS: CCDS16654.1, UniProtKB/Swiss-Prot: P01887) for hippocampus and cerebellum endogenous controls; the endogenous control for hypothalamic tissue was *Ppib* (F: 5′-TGGAGAGCACCAAGACAGACA, R: 5′-TGCCGGAGTCGACAATGAT; source sequences: AK002357, AL363449, CA321924, Consensus CDS: CCDS23301.1, UniProtKB/Swiss-Prot: P24369, UniProtKB/TrEMBL: Q9DCY1). Melting curves revealed that only one factor per primer was amplified and that there were no measurable primer-dimers. A no-template control was also run to verify that amplification only occurred in the presence of cDNA. Both B2M [20-22] and Ppib [14,23,24] have been used as controls in qRTPCR in our previous studies, and both B2M [25,26] and Ppib [27,28] have been shown to be stable controls in other work. Furthermore, there is no statistically significant difference between groups in this study in any of the tested brain regions in terms of the amplification time of either endogenous control gene (data not shown). No sex differences have been reported for these genes, and we did not find any significant difference in the amplification time for either control gene (data not shown). The total number of individual cerebellum processed was 50 (blank: XYM *N* = 7, XXM *N* = 7, XXF *N* = 6, XYF *N* = 7; E2: XYM *N* = 6, XXM *N* = 8, XXF *N* = 4, XYF *N* = 5). The total number of individual hippocampus processed was 53 (blank: XYM *N* = 6, XXM *N* = 7, XXF *N* = 6, XYF *N* = 5, E2: XYM *N* = 9, XXM *N* = 8, XXF *N* = 6, XYF *N* = 6). The total number used for the hypothalamus was 48 (6 per group).

### Western blotting

Fresh frozen brains from estradiol-treated animals were cut into 120-μM coronal sections in a cryostat and frozen on glass microscope slides, and protein was extracted from either punch of the arcuate nucleus or microdissected hypothalamus samples as described previously [16]. For protein extraction, tissue was thawed and homogenized in 10 mM Tris, 400 mM Nacl, 1 mM DTT, 1 mM EDTA, and 10% glycerol, with 10 μl/ml protease inhibitor cocktail (Sigma) and phenylmethylsulfonyl fluoride (1 mM). After centrifugation, the total lysate protein concentrations were determined by BCA protein assay (Pierce Chemical Co., Cat# 23228.). Proteins were separated on 14% polyacrylamide-SDS gels and transferred to nitrocellulose membranes. After transfer, membranes were blocked with 5% milk and incubated with an antibody for GH (1:5,000; National Hormone & Peptide Program, CA) overnight at 4°C. After rinsing, blots were incubated for 1 h with HRP-conjugated anti-rabbit IgG secondary antibody (1:10,000; Vector Laboratories) followed by detection on X-ray film (X-OMAT) with SuperSignal West Pico Chemiluminescent Substrate (Pierce Chemical Co.). The same blots were re-probed with the monoclonal antibody against β-actin at 1:10,000 (Sigma-Aldrich Corp.). The intensities of GH and β-actin bands on individual films were measured and analyzed by densitometry with ImageJ program (NIH). Levels of GH protein were normalized to those of β-actin in each sample, and the protein amount was expressed as the ratio of GH to β-actin. For the hypothalamus, 13 individual samples only from the estradiol-treated groups were used (XYM *N* = 4, XXM *N* = 3, XXF *N* = 3, XYF *N* = 3). For the analysis of the arcuate nucleus, six animals from each genotype, all estradiol treated, were assessed.

### Statistical analysis

All data were analyzed using NCSS (2001). For gene expression data, normalized gene expression was calculated using the ΔΔ*C*_t_ method [29]. Relative quantities (RQs) were log transformed and analyzed by ANOVA with sex chromosome complement, gonadal sex, and hormone treatment as factors. For protein data, two-way ANOVAs were used with genotype and gonadal sex as the two factors. Significant results were assessed by Fisher’s exact *post hoc* tests that adjust significance levels to take multiple comparisons into account.

## Results

### Estradiol increases Gh mRNA in the hippocampus and cerebellum

**Figure 1:**
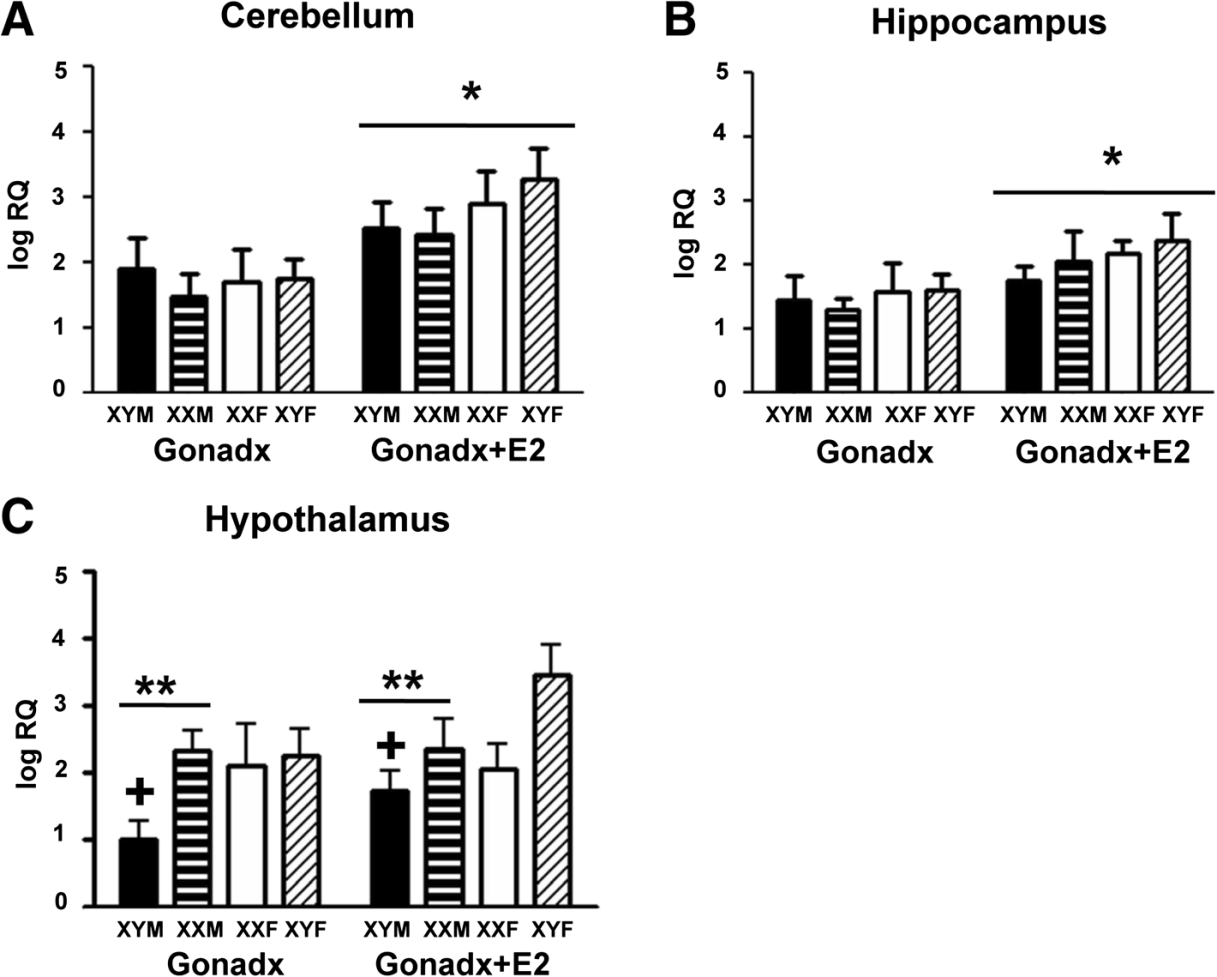
Gh mRNA in **(A)** the cerebellum, showing a significant increase in the relative quantity (RQ) of Gh mRNA in estradiol-treated animals of all genotypes; **(B)** the hippocampus, a significant effect of estradiol on Gh RQ was noted; and **(C)** the hypothalamus where effects of both sex chromosomes and gonadal sex were observed. Adult mice from the four core genotypes—XY males (black bars), XX males (horizontal striped bars), XX females (white bars), and XY females (diagonal striped bars)—were gonadectomized and treated with estradiol (E) or given empty implants. For the cerebellum: blank: XYM *N* = 7, XXM *N* = 7, XXF *N* = 6, XYF *N* = 7; E2: XYM *N* = 6, XXM *N* = 8, XXF *N* = 4, XYF *N* = 5. In the hippocampus: blank: XYM *N* = 6, XXM *N* = 7, XXF *N* = 6, XYF *N* = 5; E2: XYM *N* = 9, XXM *N* = 8, XXF *N* = 6, XYF *N* = 6. In the hypothalamus: *N* = 6 for all groups. The single asterisks indicate significant effect of estradiol (*P* < 0.05). The double asterisks indicate significant sex difference (*P* < 0.05). The plus symbols indicate mice in the XY male group which are significantly different from mice in the XX male and XY female groups (*P* < 0.05).

Estradiol significantly increased *Gh* gene expression in both the hippocampus and cerebellum. FCG mice that were treated with an estradiol implant at the time of gonadectomy had higher levels of mRNA in the cerebellum than those that were given a blank implant (Figure 1A; *F*_1,49_ = 8.61, *P* < 0.006), demonstrating for the first time that estradiol increases *Gh* mRNA levels in the cerebellum. Confirming previous results in rats [15], estradiol-treated FCG mice had higher levels of mRNA in the hippocampus than gonadectomized mice without any hormone replacement (Figure 1B; *F*_1,52_ = 5.78, *P* < 0.03). There were no main effects of gonadal sex and sex chromosome complement, nor were there any significant interactions.

### In the hypothalamus, gonadal sex and sex chromosome complement modify Gh mRNA

In the hypothalamus of FCG mice, there was a main effect of gonadal sex; males had less *Gh* expression as compared to females (Figure 1C; *F*_1,47_ = 4.41, *P* < 0.05). An interaction between gonadal sex and sex chromosome complement (*F*_1,47_ = 9.21, *P* < 0.01) was produced because XY female mice had higher *Gh* mRNA than XY male mice (*P* < 0.05). Animals given estradiol implants at the time of gonadectomy tended to have higher levels of mRNA than those that were given a blank implant at the time of gonadectomy, but this effect was not significant (*F*_1,47_ = 2.69, *P* = 0.11).

### Sex chromosome complement modifies GH protein in the whole hypothalamus of estradiol-treated animals

**Figure 2:**
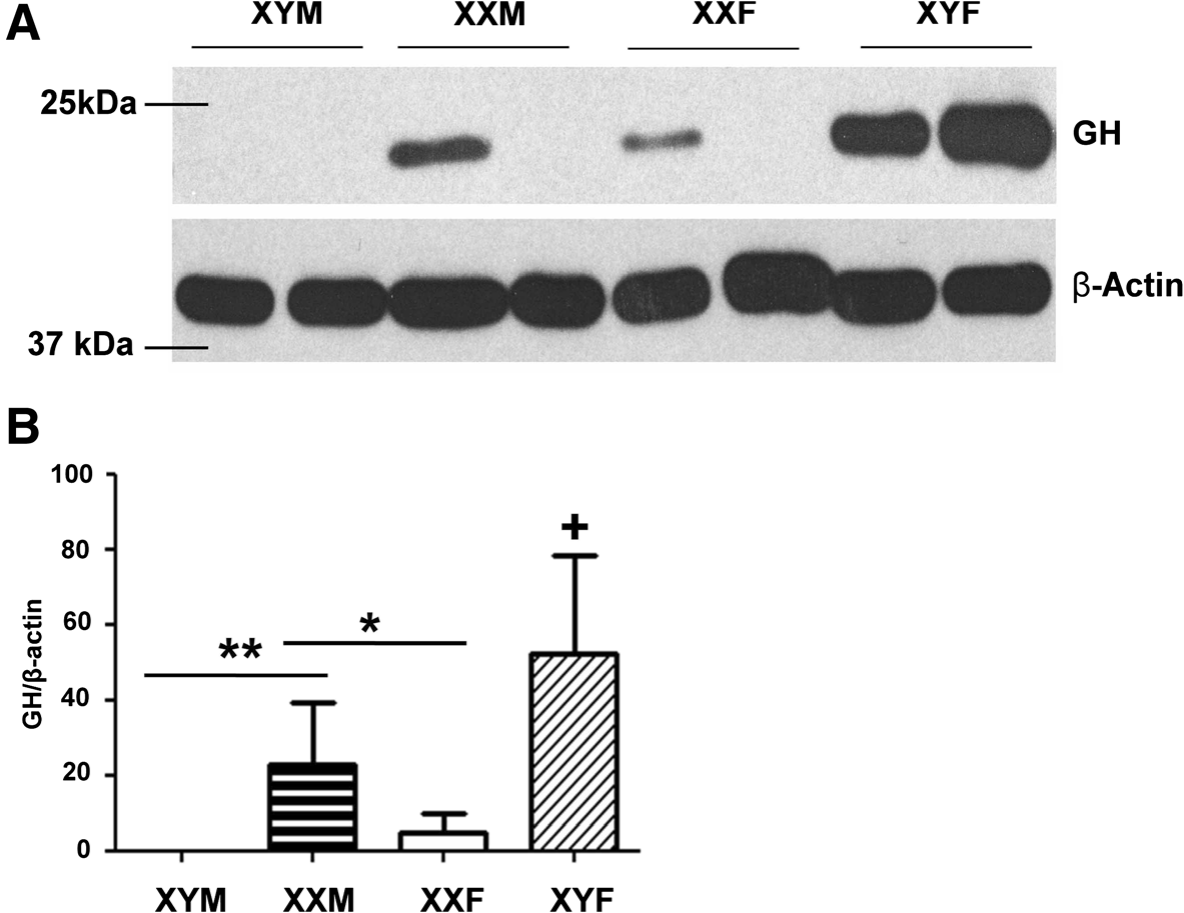
GH protein in the hypothalamus. **(A)** A representative blot is shown. **(B)** Densitometry data from the blots is presented. Adult mice from the four core genotypes—XY males (XYM *N* = 4, black bars), XX males (XXM *N* = 3, horizontal striped bars), XX females (XXF *N* = 3, white bars), and XY females (XYF *N* = 3, diagonal striped bars)—were gonadectomized and treated with estradiol (E). The single asterisk denotes significant effect of sex chromosome complement (*P* < 0.05). The double asterisks denote significant effect of gonadal sex (*P* < 0.05). The plus symbol denotes XY females which are significantly different from all other groups (*P* < 0.05).

In the hypothalamus, we noted an interaction between gonadal sex and sex chromosomes. Female mice had higher Gh protein than males, and XY animals had more Gh than XX mice (*F*_1,13_ = 20.04, *P* < 0.002). The amount of GH in XY female hypothalamus tissue was significantly greater than that in all other groups (*P* < 0.05). There was also a main effect of gonadal sex wherein females had more GH protein than males (Figure 2; *F*_1,13_ = 9.75, *P* < 0.02) and a main effect of sex chromosome complement (*F*_1,13_ = 7.59, *P* < 0.03) where GH levels in XY animals were greater than in XX animals.

### Sex chromosome complement modifies GH protein in the arcuate nucleus of the hypothalamus

**Figure 3:**
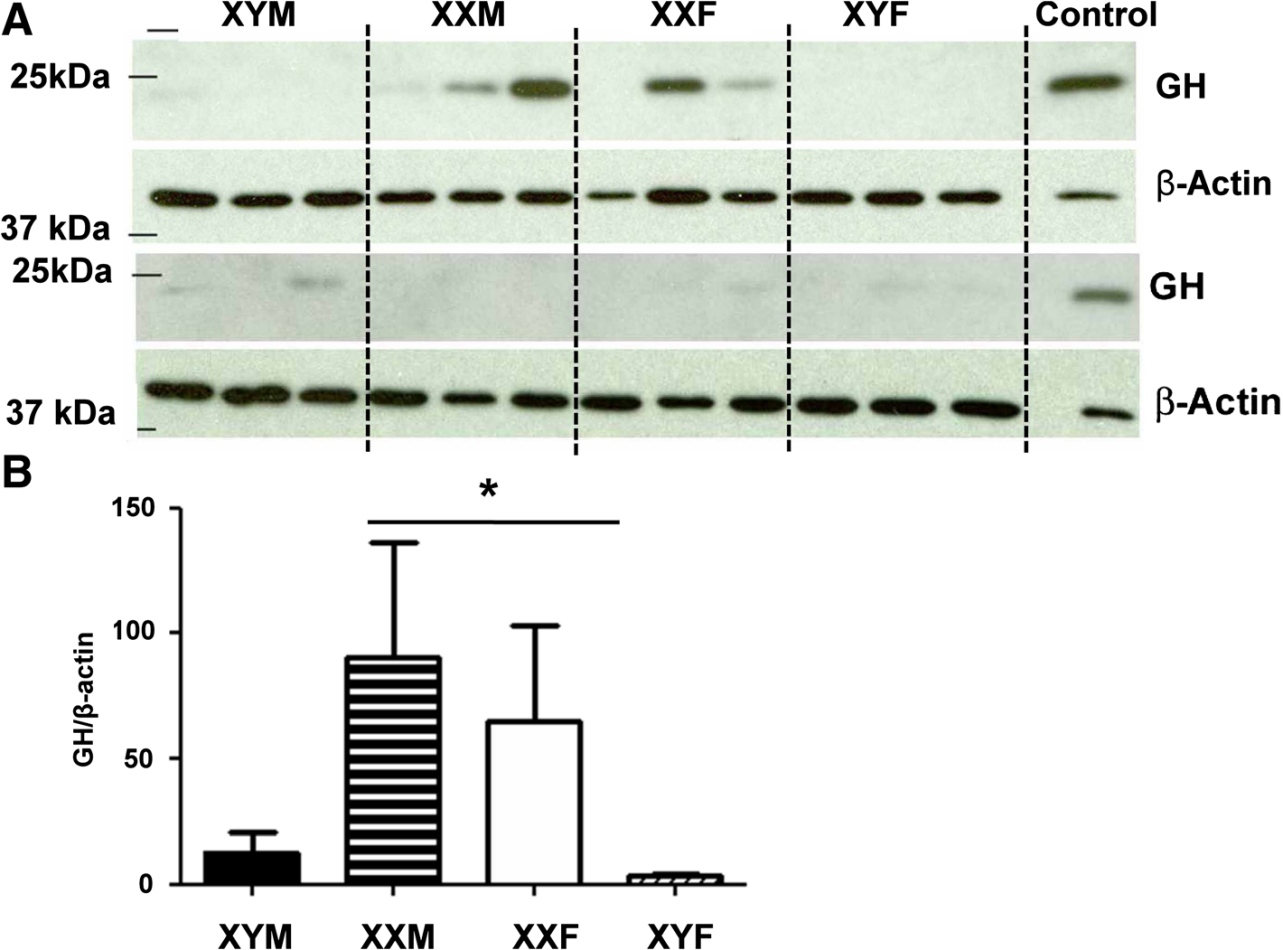
GH protein in the arcuate nucleus of the hypothalamus. **(A)** A representative blot is shown. **(B)** Densitometry data from the blots is presented. Adult mice from the four core genotypes—XY males (black bars), XX males (horizontal striped bars), XX females (white bars), and XY females (diagonal striped bars)—were gonadectomized and treated with estradiol (E). *N* = 6 in each group. The single asterisk signifies significant effect of sex chromosome complement (*P* < 0.05).

In the arcuate nucleus of the hypothalamus, we also noted an effect of sex chromosomes in estradiol-treated gonadectomized FCG mice (Figure 3; *F*_1,20_ = 5.45, *P* < 0.04). In this nucleus, XX mice had higher levels of GH protein than XY mice, regardless of gonadal sex (*P* < 0.05). No gonadal sex effect or interactions were present.

## Discussion

Our data show that estradiol treatment increases *Gh* gene expression in the cerebellum and hippocampus with a marginal effect in the hypothalamus, and that sex chromosome complement is correlated with *Gh* mRNA in the hypothalamus. Sex chromosome complement affects GH protein in the whole hypothalamus as well as the arcuate nucleus. Previous studies conducted by our lab showed that *Gh* mRNA is elevated in response to estradiol in the normal C57BL/6 female mouse hypothalamus, but not in males [16]. In combination with the current finding that GH protein is dependent on sex chromosomes in the whole hypothalamus as well as the arcuate nucleus, we suggest that estradiol and sex chromosome genes both regulate hypothalamic *Gh* gene expression.

Evidence for direct action of estradiol on GH, and factors that mediate GH release, has existed for many years. In rats, neurons containing GH-releasing factor (GHRF) in the hypothalamus and GH cells in the pituitary co-express estrogen receptors [30]. In mice, agonist binding to estrogen receptors alpha and beta induces expression of *Gh* in the pituitary [31]. Moreover, estradiol increases *Gh* mRNA in the mPOA and arcuate nucleus of female mice, and the estrogen receptor antagonist tamoxifen blocks this effect [16]. Estradiol treatment also stimulates GH secretion in humans, increasing the amount of GH per pulse twofold [4,5]. In addition, GH receptor expression and GH binding increase in an estradiol-dependent manner in rat and human bone cell lines [32], and this relationship between estradiol and GH extends to the liver [33] and ovaries [34]. It is clear from these previous findings, and more so now with the addition of this study, that estradiol has an important regulatory effect on *Gh* mRNA and GH protein. While a relationship in the hypothalamus and rat hippocampus was known, we now show that this is true in multiple brain regions, which we expect to have widespread implications.

Given the presence of sex differences in *Gh* expression across several brain areas, we speculate that GH may regulate sexually dimorphic processes throughout the body. For example, *Gh* gene expression in the brain correlates with increased food consumption and body weight in mice [13,14] and has been implicated in feeding behavior and energy homeostasis [35,14]. In a previous study using FCG mice, XY mice, independent of gonadal sex, were more sensitive to the reward of palatable food intake [36], which may be representative of the differences that we saw in GH. This would likely be due to actions in the hypothalamus, particularly in the arcuate nucleus, which has a known role in metabolism and feeding behavior. In addition, GH deficiency has been linked with cognitive impairment [16,35,37], and previous research has shown that *Gh* expression increases in the rat hippocampus following repeated learning in response to eye-blink conditioning [38]. In mice, infusion of GH into the hippocampus changes expression of immediate early genes and induces spontaneous locomotion, grooming, and anxiety-like behavior [39]. Consistent with observations in rat, our data in mice show that *Gh* is expressed in the hippocampus in a hormone-dependent manner; this may be an important aspect of learning and memory mediation by *Gh*. The cerebellum is also notably important for motor function and learning [40], and we show that estradiol regulates *Gh* levels in this region too.

Pituitary GH release is sexually dimorphic [41], and estradiol is an important activating factor in this sex difference [42]. When thinking in terms of sexual dimorphism, it is imperative to consider that estradiol and other hormones are often the only contributing element. However, steroid hormones also commonly function in conjunction with, and in addition to, sex chromosome complement, which is further reinforced by the data in this study. Sex chromosome complement is an important factor for sex differences and is implicated in sexually dimorphic outcomes of aggression [43], social interaction [43-45], and body weight [46-48]. Our data show the significance of sex chromosome complement for *Gh* gene expression and GH protein in the hypothalamus and the arcuate nucleus of the hypothalamus. The use of the FCG mice in these experiments demonstrates that the sex differences in these regions are not caused exclusively by estradiol, instead suggesting that these two principal mechanisms (estradiol and sex chromosomes) are acting together. We have observed a similar interaction in previous work. An interaction between ERα and sex chromosomes regulates calbindin gene expression in the prefrontal cortex and cerebellum of mice [49]. Notably, *Gh* and calbindin are autosomal genes, and the mechanism driving sex chromosome complement influence on autosomal gene expression is unclear. One strong possibility is that genes on the X chromosome that escape X inactivation modulate autosomal gene expression. Consistent with this proposition, *Gh* expression in the POA positively correlates with the combined levels of two X chromosome genes: *Kdm5c* and *Kdm6a*, which encode lysine demethylases [14]. KDM5c (lysine demethylase 5c) specifically demethylates Histone 3 Lysine 4 (H3K4) marks, acting as a transcriptional repressor. KDM6a demethylates Histone 3 Lysine 27 (H3K27) and is a transcriptional activator of gene transcription. Both *Kdm5c* and *Kdm6a* escape X inactivation dosage compensation and therefore have higher expression levels in XX females (as well as FCG XX males) [50,51]. These X chromosome genes may modulate estradiol and sex chromosome actions in the hypothalamus.

An intriguing aspect of these data is the substantial variance in the levels of *Gh* mRNA and GH protein within individuals in the genotypes that have the highest amounts of GH. This same variability was noted in our first two studies in normal C57BL/6 mice and in the aneuploid Y* mutant line [16,14]. Secretion of GH from the pituitary is pulsatile throughout the day [3-5]; therefore, we speculate that GH may be likewise produced and released via an ultradian rhythm. Moreover, this hypothesis suggests that GH produced in the hypothalamus is involved in the regulation of pituitary GH. If such a role for neural GH exists, gene and protein expression of GH in neural cells could respond to feedback from peripheral GH concentrations commensurate with secretory pulsatility. The pulsatility in GH secretion from the pituitary is sexually dimorphic, and X chromosome dosage may play a role in the dimorphism [41] in addition to regulation by hormones. The mice in our study were gonadectomized and received an estradiol or blank implant. Thus, endogenous fluctuations of circulating estradiol do not account for the individual variation we have noted both in GH protein and mRNA.

These data indicate that mRNA levels measured in the whole hypothalamus are comparable to whole hypothalamic protein levels. However, the direction of the relationship between amounts of GH and the sex chromosomes, with XYF hypothalami containing the greatest amounts of GH and message, is in stark contrast to the data we collected in the arcuate and mPOA in normal C57BL/6 mice [8]. In that study, females (XX) had more *Gh* mRNA than males (XY). In the aneuploidy (XY* model) mouse mutant, we measured Gh mRNA in mPOA and found a significant positive relationship between levels of *Gh* and numbers of X chromosomes [9]. Thus, our protein data from the arcuate nucleus in the FCG mice is in agreement with the data collected in normal mice. The fact that the entire hypothalamus and the arcuate are regulated differently by sex chromosome complement suggests cellular differences in the transcriptional regulation of GH. Cellular phenotypes in the hypothalamus are not uniform, and one hypothesis is that estrogen receptor alpha, which is co-expressed in nearly all GH-positive neurons in the arcuate nucleus and mPOA [16], interacts with sex chromosome genes that escape X inactivation.

## Conclusions

We have shown that *Gh* gene expression is influenced by estradiol in the cerebellum and hippocampus. In the hypothalamus, an interaction between estradiol and sex chromosome complement was produced by the differences in XY male and female mice, with males having the lowest GH protein and mRNA and females having the greatest amounts. In estradiol-treated mice, GH protein levels in the arcuate nucleus were dependent on sex chromosome complement since XX mice of both gonadal sexes had higher levels than XY mice. This work draws attention to sex differences of *Gh* gene expression and GH protein in previously unexplored areas of the brain. Future research should focus more on region-specific roles for *Gh* in physiological processes and behavior, as well as which X chromosome genes have a role in autosomal gene expression and how they act in conjunction with steroid hormones.
